# Optimal use of tranexamic acid for total hip arthroplasty: A network meta-analysis

**DOI:** 10.1371/journal.pone.0206480

**Published:** 2018-10-31

**Authors:** Byung-Ho Yoon, Tae-young Kim, Young Seung Ko, Young-Kyun Lee, Yong-Chan Ha, Kyung-Hoi Koo

**Affiliations:** 1 Department of Orthopaedic Surgery, Inje University College of Medicine, Seoul Paik Hospital, Seoul, Korea; 2 Department of Orthopaedic Surgery, Konkuk University Medical Center, School of Medicine, Konkuk University, Seoul, Korea; 3 Department of Orthopaedic Surgery, Seoul National University Bundang Hospital, Seongnam, South Korea; 4 Department of Orthopaedic Surgery, Chung-Ang University College of Medicine, Seoul, South Korea; Universita degli Studi di Firenze, ITALY

## Abstract

**Background:**

Tranexamic acid (TXA) has been demonstrated to minimize blood loss after total hip arthroplasty. There are three main routes: intravenous (IV), intra-articular (topical), and combined (IV and topical) but little consensus support which is most effective and safe. We performed network meta-analysis.to assess the comparative efficacy and safety of three different administration routes of TXA.

**Methods:**

Twenty-five randomized controlled trials (RCT) were evaluated. Interventions were classified as: combined, IV multiple, IV single, topical and placebo. The primary outcome was effectiveness (transfusion rate, total blood loss, and total drain out). The secondary outcome was safety, based on the incidence of deep venous thrombosis (DVT) and pulmonary embolism (PE).

**Results:**

A total of 2227 patients were included in the 5 categories: 564 IV single, 319 IV multiple, 398 topical, 120 combined, and 826 placebo. A network meta-analysis identified the most effective interventions in terms of reducing the need for transfusion as follows: combined = 98.2%, IV single = 54.0%, IV multiple = 78.6%, topical = 66.1%, placebo = 0.0%. Compared with placebo, the IV single, IV multiple, topical, and combined interventions showed no difference in the rate of occurrence of DVT and PE.

**Conclusions:**

A network meta-analysis indicated that combined administration of TXA (IV and topical) was effective in reducing the transfusion rate after hip arthroplasty compared with IV or topical alone. As no high-risk patients were evaluated in the RCTs, it is not known whether the combined method is safer for patients susceptible to DVT or PE.

## Introduction

Total hip arthroplasty (THA) provides excellent results in patients with end-stage hip disease.[1] However, blood loss during the procedure can be as much as 500 to 2000 ml, and the proportion of patients requiring transfusion range from 16% to 20%.[2, 3] To reduce blood loss and the need for transfusion, several prophylactic blood management protocols have been developed. [4]

Tranexamic acid (TXA), a synthesized anti fibrinolytic agent, inhibits the conversion of plasminogen to plasmin by competitively binding to the lysine-binding sites of plasminogen.[5] Thus, it effectively reduces postoperative bleeding by inhibiting fibrinolysis and clot degradation. Currently, six pairwise meta-analyses of clinical trials using THA have shown that TXA applied topically or intravenously can decrease blood loss and transfusion rate without an increased risk of complications.[6–11] However, the optimal administration route for TXA to achieve efficacy and safety after THA is still controversial. Methods of application include topical, intravenous (IV), and combined IV and topical. It is unclear which of these methods is most effective to reduce total blood loss and to reduce total drain out.

Traditional pair-wise meta-analysis can only address questions regarding pairs of treatments; therefore, they cannot determine which of the several interventions is most effective. A network meta-analysis (NMA) is a useful statistical method for comparing the efficacy of multiple therapies. Joint analysis of the data within a network framework allows novel inferences on treatment comparisons by combining direct and indirect evidence of all relative treatment effects with a common comparator. Combining the estimates of direct and indirect comparisons provides mixed estimates with maximum power and produces a relative ranking of all treatments for the studied outcome.[12]

We conducted an NMA to elucidate which method is most effective and safe during THA. We have also included up-to-date studies that were not used in previous meta-analyses in order to strengthen the evidence. The comparative efficacy and safety of TXA were investigated by comparing (1) transfusion rate, (2) total blood loss, (3) total drainage, (4) deep vein thrombosis (5), and pulmonary embolism.

## Materials and methods

### Search strategy

This study was conducted and reported based on guidelines and recommendations for network meta-analysis (S1 Table).[12] Searches of PubMed-Medline, Embase, and the Cochrane Library were performed in April 2017 using key terms (Hip OR arthroplasty OR replacement AND tranexamic acid). The detailed search strategies in each data base are presented in S2 Table. All trials had to be randomized, double-blinded, and controlled to ensure a minimum high level of quality, and study subjects with total hip arthroplasties. We also checked the reference lists of all potentially eligible studies and review papers to find any additional relevant publications.

### Selection of studies

In the first stage of the study selection, two independent reviewers (YBH, KTY) first screened the titles and abstracts in order to identify relevant articles. To be included, patients had to have undergone total hip arthroplasty; no restriction was set regarding underlying causes or type of implant. The type of intervention had to include administration by IV, topical, or combined routes, or use of placebo. Outcome had to be measured using at least one of the following: rate of transfusion, total blood loss, total drain out, the incidence of deep venous thrombosis and pulmonary embolism after THA. Studies that used other strategies to prevent blood loss were excluded, as were studies with less than 10 subjects or incomplete data, prospective non-randomized studies, and non-clinical studies (laboratory, animal, protocol, basic science, review). The language was restricted to English. When a published, updated study involving the same cohort of patients was identified, only the latest update was included in the analysis.

### Data extraction

With respect to IV intervention, the protocols differed for each study. We classified them into two groups: ‘IV single’, in which TXA was injected only once; and ‘IV multiple’, in which TXA was injected more than twice. ‘IV single’ included single doses of: 10 mg/kg, 15 mg/kg, 1 g, and 3 g. ‘IV multiple’ included dual doses of: 10 mg/kg, 15 mg/kg bolus and infusion, a twice dose of 2 g, and a triple dose of 3 g. ‘Combined’ included IV 15 mg/kg bolus and 1 g topical injection; IV 1g bolus and 2g topical injection. The interventions were classified into five categories: IV single, IV multiple, topical, combined, and placebo.

For every eligible study, the following data were extracted and entered into a spread sheet by the two reviewers: the first author, the year of publication, the number of patients, enrollment period, the mean age at the time of the operation, TXA treatment (route of administration, dose, bolus or continuous, single or multiple injection), the criteria for transfusion, the use of thromboprophylaxis, and the length of follow-up.

### Quality assessment

All included articles were individually evaluated for risk of bias by two authors (YBH, KTY) respectively using the risk of bias assessment tool, as described in the Cochrane Handbook for Systematic Reviews of Interventions.

### Data synthesis and analysis

Two types of statistical analyses were performed for the extracted data. The first was a pair-wise meta-analysis of the included studies. For each study, we calculated the relative risks with a 95% confidence interval (CI) by using crude 2×2 tables, whenever possible, from the comparative studies[13]. The Mantel–Haenzel method was used to calculate the odds-ratio if there were zero values in any cell count in a table.[14] To evaluate the presence of clinical heterogeneity within pairwise comparisons, taking a conservative approach, heterogeneity between comparable studies was tested with chi-square (χ^2^) and I^2^ tests. *P* > 0.1 and I^2^ < 50% were the established criteria to determine the statistical heterogeneity.

A network meta-analysis was then conducted to determine comparative efficacy and safety. This type of analysis allowed us to utilize results from the comparison of two interventions with the same third intervention for indirect comparisons. For a given comparison, such as A versus B, direct evidence is provided by studies that compare two treatments directly (IV versus placebo) as in a standard direct pair-wise meta-analysis. In addition, indirect evidence for A versus B can be provided if studies that compare A versus C and B versus C are analyzed jointly (e.g., IV versus placebo studies and topical versus placebo studies can allow indirect comparison of IV versus topical via the use of placebo). All three interventions (IV, topical, and combined) had trial data compared with placebo. NMA aims to combine the direct and indirect evidence into a single-effect size and thereby increases the precision of the comparison. Valid results from NMA depend on the evidence network being internally consistent; direct and indirect sources of evidence should be in an agreement. A test of inconsistency was completed by using the loop-specific approach to evaluate heterogeneity and consistency of the network. To rank the treatments according to safety or efficacy, we planned to use the surface under the cumulative ranking (SUCRA) probabilities, which are expressed as percentages of each intervention to an imaginary intervention that is always the best without uncertainty. For example, if combined administration had an 80% probability of superiority, this would be interpreted as follows: in 80% of the simulation runs, combined administration had the highest number of success rates out of all treatment modalities. Corresponding 95% credible intervals (CrIs) were obtained using the 2.5^th^ and 97.5^th^ percentiles of the posterior distribution.

All analyses were performed using STATA (version 14.0; Stata Corporation, College Station, TX, USA) with the “mvmeta” command[15] and self-programmed STATA routines, as described in Chaimani et al.[16] This study was exempted from institutional review board review because it did not involve human subjects.

### Sensitivity analysis and publication bias

A sensitivity analysis was conducted by including only the studies that included transfusions with a strict transfusion strategy (hemoglobin level < 8 g/dl or < 9 g/dl in older patients with cardiopulmonary disease). In addition, we compared the overall results after excluding two studies[17, 18] that used “single 3 g” and “triple 3 g” treatments to evaluate whether the results were particularly influenced by studies using high-dose IVs. Finally the overall results were compared by excluding each one study that included combined treatment [19, 20] from the pool of the twenty-five studies. The publication bias for each complication was assessed visually by the adjusted network funnel plot.

## Results

### Description of included studies

**Fig 1:**
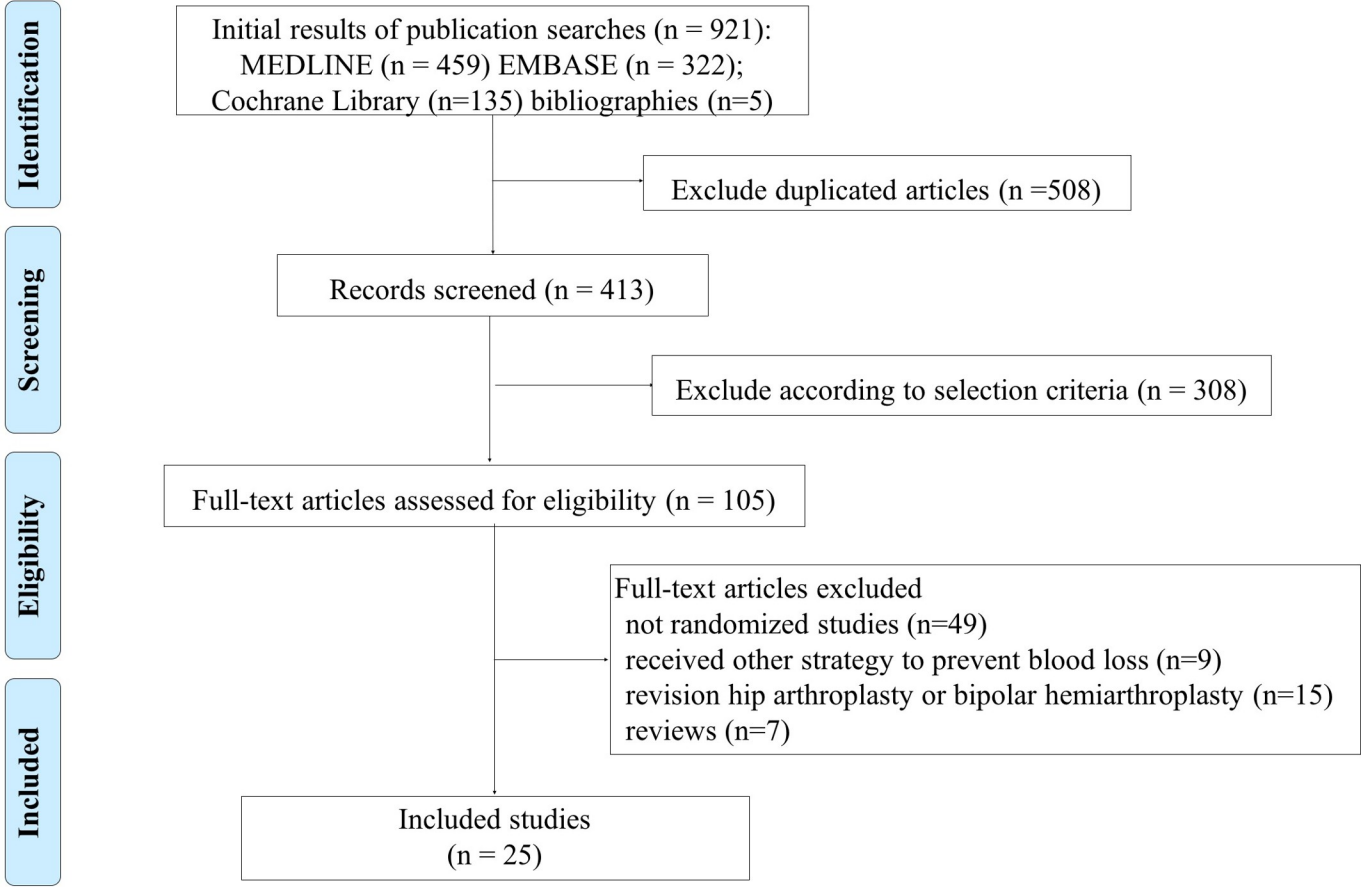
PRISMA Flow diagram details the process of relevant clinical study selection.

**Fig 2:**
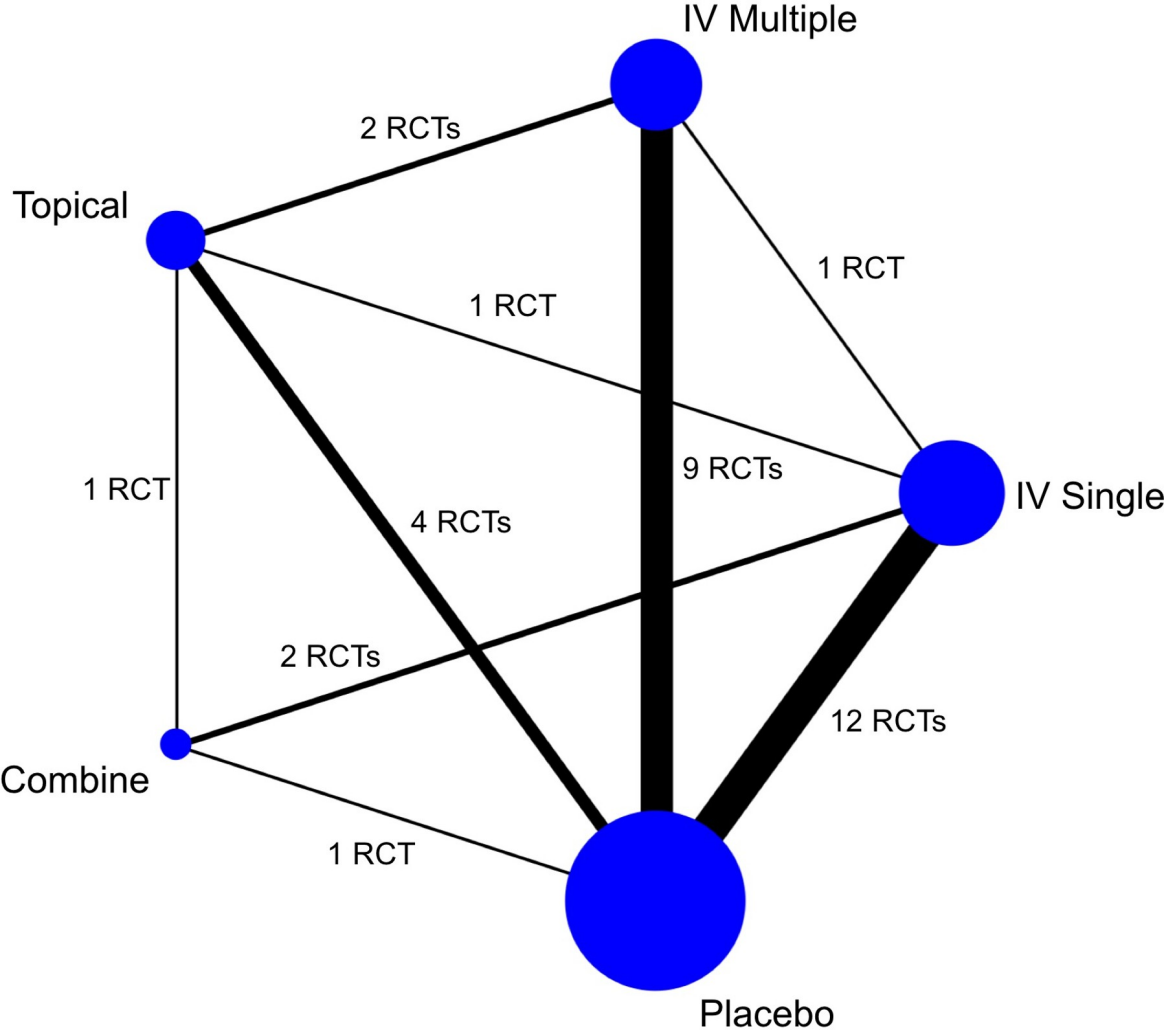
Network plot depicting the direct evidence used in the network meta-analysis, which included 25 randomized controlled trials, and 33 direct comparisons due to four multi-arm trials. The circle size is proportional to the number of patients, and line thickness is weighted proportionally to the number of trials that assess the comparison.

We identified 25 RCTs. Twenty-one studies compared two interventions: 9 IV single vs placebo[21–29], 8 IV multiple vs placebo[17, 30–36], 3 compared topical application vs placebo[37–39], and 1 IV multiple vs topical.[40] Four studies had three comparative groups: IV single vs IV multiple vs placebo[41], IV single vs topical vs placebo[18], IV single vs combined vs placebo[19], and IV single vs topical vs combined.[20] A summary of the studies from a primary search of the databases to final inclusion or exclusion is displayed in the flowchart (Fig 1). A total of 2227 patients were included in the NMA: 564 IV single, 319 IV multiple, 398 topical, 120 combined, and 826 placebo. The majority of comparisons were between IV and placebo, but all direct comparisons between interventions were found, as can be seen in the network diagram (Fig 2, S1 Fig). When we integrated the evidence from all trials into the NMA, we found no inconsistency between direct and indirect evidence in all interventions. The characteristics of the included studies are summarized in Table 1.

**Table 1:** Characteristics of included individual studies.

	Group comparison	Transfusion (patients)	Transfusion trigger	Total blood loss	Total drain out	Deep vein thrombosis	Pulonary emblism	The route and dose of tranexamic acid
Fraval 2017	B: IV twice	B: 1/50 (2%)	<8g/L	B: 1084 (440)	NA	B: 0/50 (0%)	B: 0/50 (0%)	Twice 15mg/Kg
	E: Placebo	E: 6/51 (12%)		E: 1394 (426)		E: 0/51(0%)	E: 0/51(0%)	
Barrachina 2016	A: IV single	A: 8/35 (23%)	<8.5g/L	A: 1377 (689)	NA	A: 1/35(3%)	NA	Single 15mg/Kg
	B: IV twice	B: 4/36 (11%)		B: 1308 (641)		B: 1/36 (3%)		Twice 10mg/Kg
	E: Placebo	E: 14/37 (39%)		E: 2215 (1136)		E: 2/37 (5%)		
Xie 216	A: IV single	A: 3/70 (4%)	<7g/L	A: 878 (686)	NA	A: 1/70 (1%)	A: 0/70 (0%)	Single 1.5g
	C: Topical	C: 4/70 (6%)		C: 905 (238)		C: 0/70 (0%)	C: 0/70 (0%)	Topical 3g
	D: Combine	D: 0/70 (0%)		D: 777 (189)		D: 2/70 (3%)	D: 0/70 (0%)	Combine 3g
Na 2016	A: IV single	A: 2/29 (7%)	<8g/L	NA	NA	NA	NA	Single 10mg/Kg
	E: Placebo	E: 5/26 (19%)						
North 2016	B: IV twice	B: 8/70 (11%)	<7g/L	B: 1195 (486)	NA	B: 0/70 (0%)	B: 1/70 (%)	Twice 2g
	C: Topical	C: 12/69 (17%)		C: 1443 (563)		C: 0/69 (0%)	C: 0/69 (0%)	Topical 2g
Wang 2016	A: IV single	A: 9/81(11%)	<7g/L	NA	NA	A: 1/81(1%)	A: 0/81(0%)	Single 10mg/Kg or Single 15mg/Kg
	E: Placebo	E: 10/38 (26%)				E: 0/38 (0%)	E: 0/38 (0%)	
Yi 2016	A: IV single	A: 8/50 (16%)	<7g/L	A: 1003 (367)	A:127 (92)	A: 2/50 (4%)	A: 0/50 (0%)	Single 15mg/Kg
	D: Combine	D: 1/50 (2%)		D: 836 (344)	D:127 (114)	D: 2/50 (4%)	D: 0/50 (0%)	Combine 3g
	E: Placebo	E: 19/50 (38%)		E: 1221 (386)	E: 244 (146)	E: 1/50 (2%)	E: 0/50 (0%)	
Hsu 2015	B: IV twice	B: 2/30 (7%)	<8g/L	NA	NA	B: 0/30 (0%)	B: 0/30 (0%)	Twice 2g
	E: Placebo	E: 9/30 (30%)				E: 0/30 (0%)	E: 0/30 (0%)	
Wei 2014	A: IV single	A: 6/101 (6%)	<9g/L	A: 959 (422)	NA	A: 1/101 (1%)	A: 0/101 (0%)	Single 3g
	C: Topical	C: 6/102 (6%)		C: 963 (421)		C: 1/102 (1%)	C: 0/102 (0%)	Topical 3g
	E: Placebo	E: 26/100 (26%)		E: 1364 (279)		E: 0/100 (0%)	E:0/100(0%)	
Martin 2014	C: IA	C: 3/25 (12%)	<7g/L	NA	NA	C: 0/25 (0%)	C: 0/25 (0%)	Topical 2g
	E: Placebo	E: 5/25 (20%)				E: 0/25 (0%)	E: 0/25 (0%)	
Yue 2014	C: Topical	C: 3/52 (6%)	<7g/L	C: 346 (332)	C: 218 (90)	C: 1/52 (2%)	C: 0/52 (0%)	Topical 3g
	E: Placebo	E: 11/49 (22%)		E: 1256 (194)	E: 297 (109)	E: 0/49 (0%)	E: 0/49 (0%)	
Alshryda 2013	C: Topical	C: 10/80 (13%)	NA	C: 1617 (188)	C: 260 (188)	C: 2/80 (3%)	C: 0/80 (0%)	NA
	E: Placebo	E: 26/81 (32%)		E: 1981 (1007)	E: 389 (187)	E: 4/81 (5%)	E: 0/81 (0%)	
Lee 2013	B: IV twice	B: 9/34 (26%)	Hb loss >30%	B:647 (216)	B:439 (172)	B: 0/34 (0%)	NA	Twice 15mg/Kg
	E: Placebo	E: 20/34 (59%)		E:1326 (349)	E:1074 (287)	E: 0/34 (0%)		
Kazemi 2010	A: IV single	A: 7/32 (22%)	NA	NA	NA	A: 0/32 (0%)	A: 0/32 (0%)	Single 15mg/Kg
	E: Placebo	E: 15/32 (49%)				E: 1/32 (3%)	E: 0/32 (0%)	
Rajesparan 2009	A: IV single	A: 3/36 (8%)	NA	A:1372 (436)	NA	A: 1/36 (2%)	A: 0/36 (0%)	Single 1g
	E: Placebo	E: 10/37 (27%)		E:1683 (705)		E: 2/37 (5%)	E: 0/37 (0%)	
Claeys 2007	A: IV single	A: 1/20 (5%)	<8.5g/L	NA	A:352 (152)	A: 3/20 (15%)	NA	Single 15mg/Kg
	E: Placebo	E: 6/20 (30%)			E:524 (244)	E: 0/20 (0%)		
Johansson 2005	A: IV single	A: 8/47 (17%)	NA	A:969 (434)	A:350 (114)	NA	NA	Single 15mg/Kg
	E: Placebo	E: 23/53 (43%)		D:1324 (577)	D:528 (211)			
Niskanen 2005	A: IV single	A: 5/19 (26%)	NA	A:626 (206)	A:166 (75)	A: 0/19 (0%)	A: 0/19 (0%)	Three doses of 10mg/Kg
	E: Placebo	E: 8/20 (40%)		E:790 (293)	E:312 (126)	E: 0/20 (0%)	E: 0/20 (0%)	
Garneti 2004	A: IV single	A: 16/25 (64%)	NA	A:1443 (809)	A:411 (220)	A: 0/25 (0%)	A: 1/25 (4%)	Single 10mg/Kg
	E: Placebo	E: 14/25 (56%)		E:1340 (665)	E:353 (311)	E: 0/25 (0%)	E: 0/25 (0%)	
Lemay 2004	B: IV twice	B: 6/20 (30%)	<7g/L	B:1308 (462)	B:487 (234)	B: 0/20 (0%)	B: 0/20 (0%)	Twice 10 mg/kg (bolus and infusion)
	E: Placebo	E: 13/19 (68%)		E:1469 (405)	E:580 (290)	E: 0/19 (0%)	E: 0/19 (0%)	
Yamasaki 2004	A: IV single	A: 0/20 (0%)	NA	A:1350 (477)	A:655 (418)	A: 0/20 (0%)	NA	Single 1g
	E: Placebo	E: 0/20 (0%)		E:1667 (401)	E:890 (353)	E: 0/20 (0%)		
Husted 2003	B: IV twice	B: 2/20 (10%)	Hb loss >25%	NA	NA	B: 0/20 (0%)	B: 0/20 (0%)	Twice 10 mg/kg (bolus and infusion)
	E: Placebo	E: 7/20 (35%)				E: 0/20 (0%)	E: 0/20 (0%)	
Benoni 2001	A: IV single	A: 4/18 (22%)	<10g/L	A:759 (193)	NA	A: 0/18 (0%)	A: 0/18 (0%)	Single 10mg/Kg
	E: Placebo	E: 8/20 (40%)		E:996 (267)		E: 0/20 (0%)	E: 0/20 (0%)	
Ekbäck 2000	B: IV twice	B: 1/20 (5%)	Hct loss >27%	B:1130 (400)	NA	B: 1/20 (5%)	NA	Twice 10mg/Kg
	E: Placebo	E: 1/20 (5%)		E:1770 (523)		E: 1/20 (5%)		
Benoni 2000	B: IV twice	B: 9/20 (45%)	NA	B:550 (275)	A:440 (303)	B: 3/20 (15%)	B: 0/20 (0%)	Twice 10mg/Kg
	E: Placebo	E: 15/19 (79%)		E:500 (234)	E:450 (271)	E: 3/19 (16%)	E: 0/19 (0%)	
	Group comparison	Transfusion (patients)	Transfusion trigger	Total blood loss	Total drain out	Deep vein thrombosis	Pulonary emblism	The route and dose of tranexamic acid
Fraval 2017	B: IV twice	B: 1/50 (2%)	<8g/L	B: 1084 (440)	NA	B: 0/50 (0%)	B: 0/50 (0%)	Twice 15mg/Kg
	E: Placebo	E: 6/51 (12%)		E: 1394 (426)		E: 0/51(0%)	E: 0/51(0%)	
Barrachina 2016	A: IV single	A: 8/35 (23%)	<8.5g/L	A: 1377 (689)	NA	A: 1/35(3%)	NA	Single 15mg/Kg
	B: IV twice	B: 4/36 (11%)		B: 1308 (641)		B: 1/36 (3%)		Twice 10mg/Kg
	E: Placebo	E: 14/37 (39%)		E: 2215 (1136)		E: 2/37 (5%)		
Xie 216	A: IV single	A: 3/70 (4%)	<7g/L	A: 878 (686)	NA	A: 1/70 (1%)	A: 0/70 (0%)	Single 1.5g
	C: Topical	C: 4/70 (6%)		C: 905 (238)		C: 0/70 (0%)	C: 0/70 (0%)	Topical 3g
	D: Combine	D: 0/70 (0%)		D: 777 (189)		D: 2/70 (3%)	D: 0/70 (0%)	Combine 3g
Na 2016	A: IV single	A: 2/29 (7%)	<8g/L	NA	NA	NA	NA	Single 10mg/Kg
	E: Placebo	E: 5/26 (19%)						
North 2016	B: IV twice	B: 8/70 (11%)	<7g/L	B: 1195 (486)	NA	B: 0/70 (0%)	B: 1/70 (%)	Twice 2g
	C: Topical	C: 12/69 (17%)		C: 1443 (563)		C: 0/69 (0%)	C: 0/69 (0%)	Topical 2g
Wang 2016	A: IV single	A: 9/81(11%)	<7g/L	NA	NA	A: 1/81(1%)	A: 0/81(0%)	Single 10mg/Kg or Single 15mg/Kg
	E: Placebo	E: 10/38 (26%)				E: 0/38 (0%)	E: 0/38 (0%)	
Yi 2016	A: IV single	A: 8/50 (16%)	<7g/L	A: 1003 (367)	A:127 (92)	A: 2/50 (4%)	A: 0/50 (0%)	Single 15mg/Kg
	D: Combine	D: 1/50 (2%)		D: 836 (344)	D:127 (114)	D: 2/50 (4%)	D: 0/50 (0%)	Combine 3g
	E: Placebo	E: 19/50 (38%)		E: 1221 (386)	E: 244 (146)	E: 1/50 (2%)	E: 0/50 (0%)	
Hsu 2015	B: IV twice	B: 2/30 (7%)	<8g/L	NA	NA	B: 0/30 (0%)	B: 0/30 (0%)	Twice 2g
	E: Placebo	E: 9/30 (30%)				E: 0/30 (0%)	E: 0/30 (0%)	
Wei 2014	A: IV single	A: 6/101 (6%)	<9g/L	A: 959 (422)	NA	A: 1/101 (1%)	A: 0/101 (0%)	Single 3g
	C: Topical	C: 6/102 (6%)		C: 963 (421)		C: 1/102 (1%)	C: 0/102 (0%)	Topical 3g
	E: Placebo	E: 26/100 (26%)		E: 1364 (279)		E: 0/100 (0%)	E:0/100(0%)	
Martin 2014	C: IA	C: 3/25 (12%)	<7g/L	NA	NA	C: 0/25 (0%)	C: 0/25 (0%)	Topical 2g
	E: Placebo	E: 5/25 (20%)				E: 0/25 (0%)	E: 0/25 (0%)	
Yue 2014	C: Topical	C: 3/52 (6%)	<7g/L	C: 346 (332)	C: 218 (90)	C: 1/52 (2%)	C: 0/52 (0%)	Topical 3g
	E: Placebo	E: 11/49 (22%)		E: 1256 (194)	E: 297 (109)	E: 0/49 (0%)	E: 0/49 (0%)	
Alshryda 2013	C: Topical	C: 10/80 (13%)	NA	C: 1617 (188)	C: 260 (188)	C: 2/80 (3%)	C: 0/80 (0%)	NA
	E: Placebo	E: 26/81 (32%)		E: 1981 (1007)	E: 389 (187)	E: 4/81 (5%)	E: 0/81 (0%)	
Lee 2013	B: IV twice	B: 9/34 (26%)	Hb loss >30%	B:647 (216)	B:439 (172)	B: 0/34 (0%)	NA	Twice 15mg/Kg
	E: Placebo	E: 20/34 (59%)		E:1326 (349)	E:1074 (287)	E: 0/34 (0%)		
Kazemi 2010	A: IV single	A: 7/32 (22%)	NA	NA	NA	A: 0/32 (0%)	A: 0/32 (0%)	Single 15mg/Kg
	E: Placebo	E: 15/32 (49%)				E: 1/32 (3%)	E: 0/32 (0%)	
Rajesparan 2009	A: IV single	A: 3/36 (8%)	NA	A:1372 (436)	NA	A: 1/36 (2%)	A: 0/36 (0%)	Single 1g
	E: Placebo	E: 10/37 (27%)		E:1683 (705)		E: 2/37 (5%)	E: 0/37 (0%)	
Claeys 2007	A: IV single	A: 1/20 (5%)	<8.5g/L	NA	A:352 (152)	A: 3/20 (15%)	NA	Single 15mg/Kg
	E: Placebo	E: 6/20 (30%)			E:524 (244)	E: 0/20 (0%)		
Johansson 2005	A: IV single	A: 8/47 (17%)	NA	A:969 (434)	A:350 (114)	NA	NA	Single 15mg/Kg
	E: Placebo	E: 23/53 (43%)		D:1324 (577)	D:528 (211)			
Niskanen 2005	A: IV single	A: 5/19 (26%)	NA	A:626 (206)	A:166 (75)	A: 0/19 (0%)	A: 0/19 (0%)	Three doses of 10mg/Kg
	E: Placebo	E: 8/20 (40%)		E:790 (293)	E:312 (126)	E: 0/20 (0%)	E: 0/20 (0%)	
Garneti 2004	A: IV single	A: 16/25 (64%)	NA	A:1443 (809)	A:411 (220)	A: 0/25 (0%)	A: 1/25 (4%)	Single 10mg/Kg
	E: Placebo	E: 14/25 (56%)		E:1340 (665)	E:353 (311)	E: 0/25 (0%)	E: 0/25 (0%)	
Lemay 2004	B: IV twice	B: 6/20 (30%)	<7g/L	B:1308 (462)	B:487 (234)	B: 0/20 (0%)	B: 0/20 (0%)	Twice 10 mg/kg (bolus and infusion)
	E: Placebo	E: 13/19 (68%)		E:1469 (405)	E:580 (290)	E: 0/19 (0%)	E: 0/19 (0%)	
Yamasaki 2004	A: IV single	A: 0/20 (0%)	NA	A:1350 (477)	A:655 (418)	A: 0/20 (0%)	NA	Single 1g
	E: Placebo	E: 0/20 (0%)		E:1667 (401)	E:890 (353)	E: 0/20 (0%)		
Husted 2003	B: IV twice	B: 2/20 (10%)	Hb loss >25%	NA	NA	B: 0/20 (0%)	B: 0/20 (0%)	Twice 10 mg/kg (bolus and infusion)
	E: Placebo	E: 7/20 (35%)				E: 0/20 (0%)	E: 0/20 (0%)	
Benoni 2001	A: IV single	A: 4/18 (22%)	<10g/L	A:759 (193)	NA	A: 0/18 (0%)	A: 0/18 (0%)	Single 10mg/Kg
	E: Placebo	E: 8/20 (40%)		E:996 (267)		E: 0/20 (0%)	E: 0/20 (0%)	
Ekbäck 2000	B: IV twice	B: 1/20 (5%)	Hct loss >27%	B:1130 (400)	NA	B: 1/20 (5%)	NA	Twice 10mg/Kg
	E: Placebo	E: 1/20 (5%)		E:1770 (523)		E: 1/20 (5%)		
Benoni 2000	B: IV twice	B: 9/20 (45%)	NA	B:550 (275)	A:440 (303)	B: 3/20 (15%)	B: 0/20 (0%)	Twice 10mg/Kg
	E: Placebo	E: 15/19 (79%)		E:500 (234)	E:450 (271)	E: 3/19 (16%)	E: 0/19 (0%)	

IV: intravenous, NA: non-available, Hb: hemoglobin, Hct: hematocrit

### Need for transfusion

**Fig 3:**
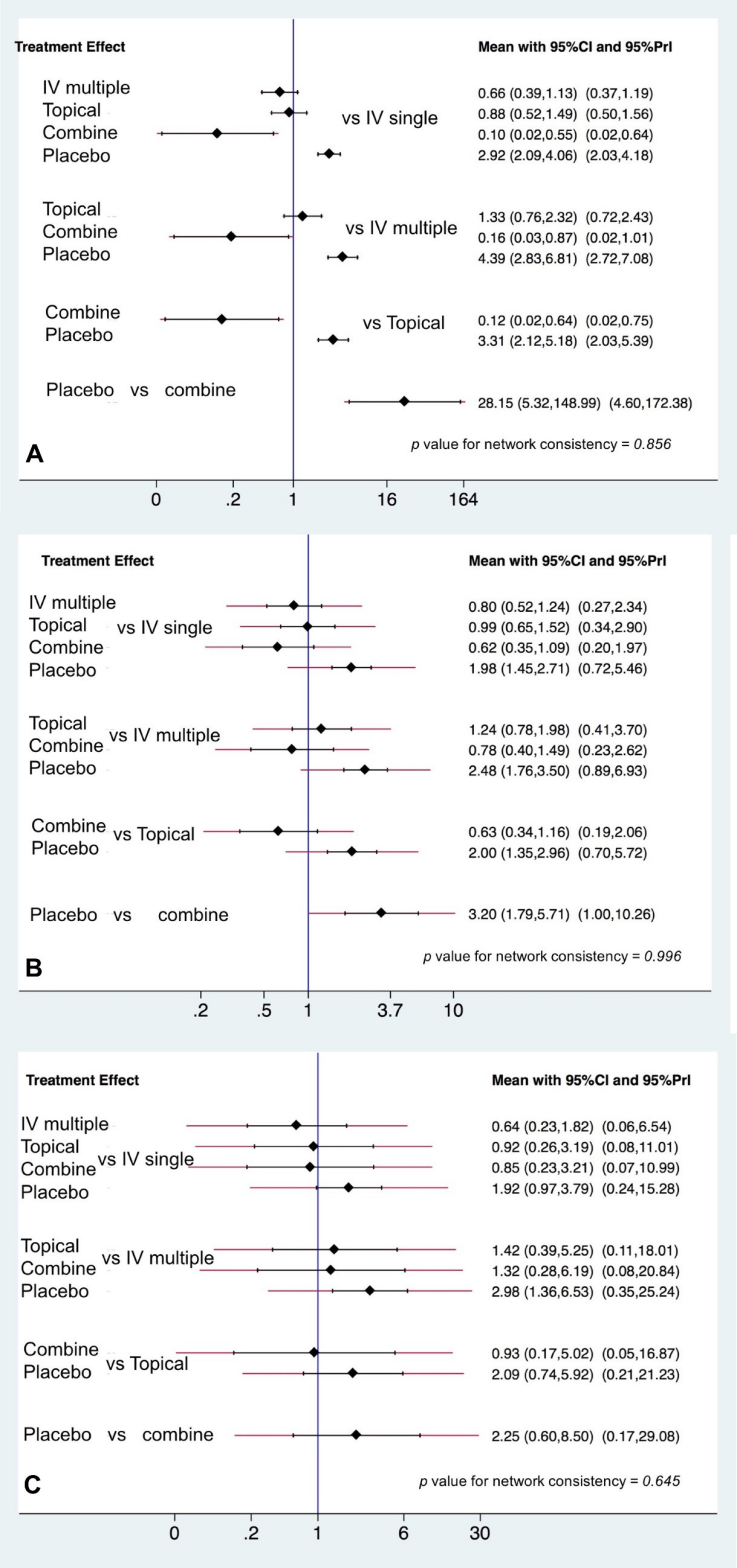
The interval plot of the odds ratio for the efficacy by (A) transfusion rate, (B) total blood loss, and (C) total drain out.

**Fig 4:**
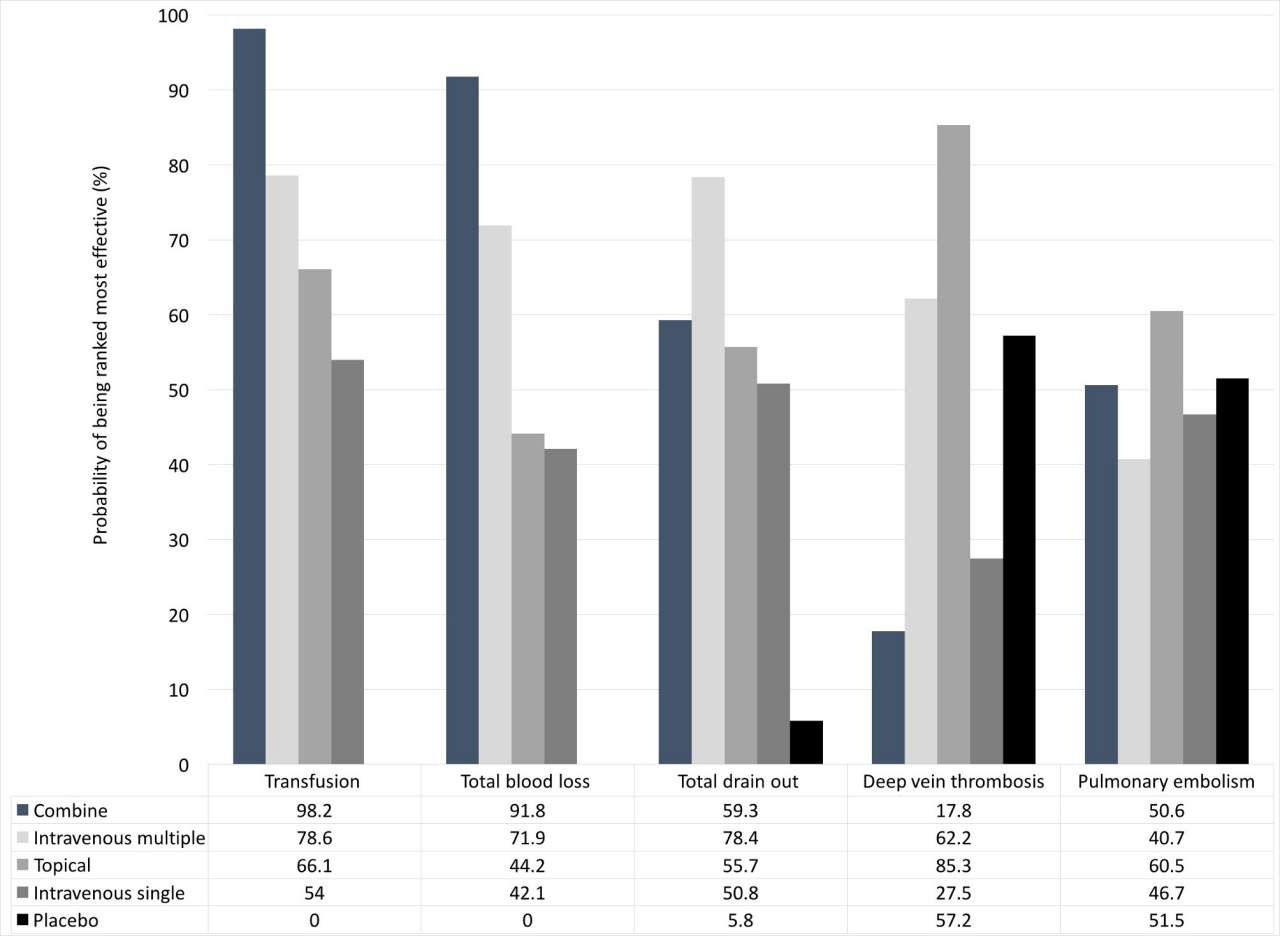
Rankogram of five interventions for each outcome.

Blood transfusion data were provided in all 25 studies. All four interventions effectively reduced transfusion compared with placebo; Combine (odds ratio (OR) = 0.04, 95% CI: 0.01–0.19, p<0.001), IV multiple (OR = 0.23, 95% CI: 0.15–0.35, p<0.001), topical use (OR = 0.30, 95% CI: 0.19–0.47, p<0.001), and IV single (OR = 0.34, 95% CI: 0.25–0.48, p<0.001). The combined method of TXA use significantly reduced the transfusion rate compared with IV single (OR = 0.10, 95% CI: 0.02–0.55, *p* = 0.008); IV multiple (OR = 0.16, 95% CI: 0.03–0.87, *p* = 0.034); and topical use (OR = 0.12, 95% CI: 0.02–0.64, *p* = 0.014) (Fig 3A). But no significant difference was observed across three interventions (IV single, IV multiple, and topical). The network meta-analysis showed the following probabilities ranked as the most effective intervention with 95% CrIs: combined = 98.2%, IV multiple = 78.6%, topical = 66.1%, IV single = 54.0%, and placebo = 0.0% (Fig 4).

### Total blood loss and total drain out

Eighteen trials[17–21, 23, 24, 27, 29–32, 35–38, 40, 41] involving a total of 1799 patients reported data for total blood loss. All interventions significantly reduced total blood loss compared with placebo; however, no difference was observed across the four non-placebo interventions (Fig 3B). Eleven trials[17, 19, 22–24, 29, 30, 35–38] with 827 patients reported data for total drain loss. There was no significant difference across four of the interventions (Fig 3C). The ranking of the five interventions with regards to total blood loss and drain out is summarized in Fig 4.

### Deep vein thrombosis and pulmonary embolism

**Fig 5:**
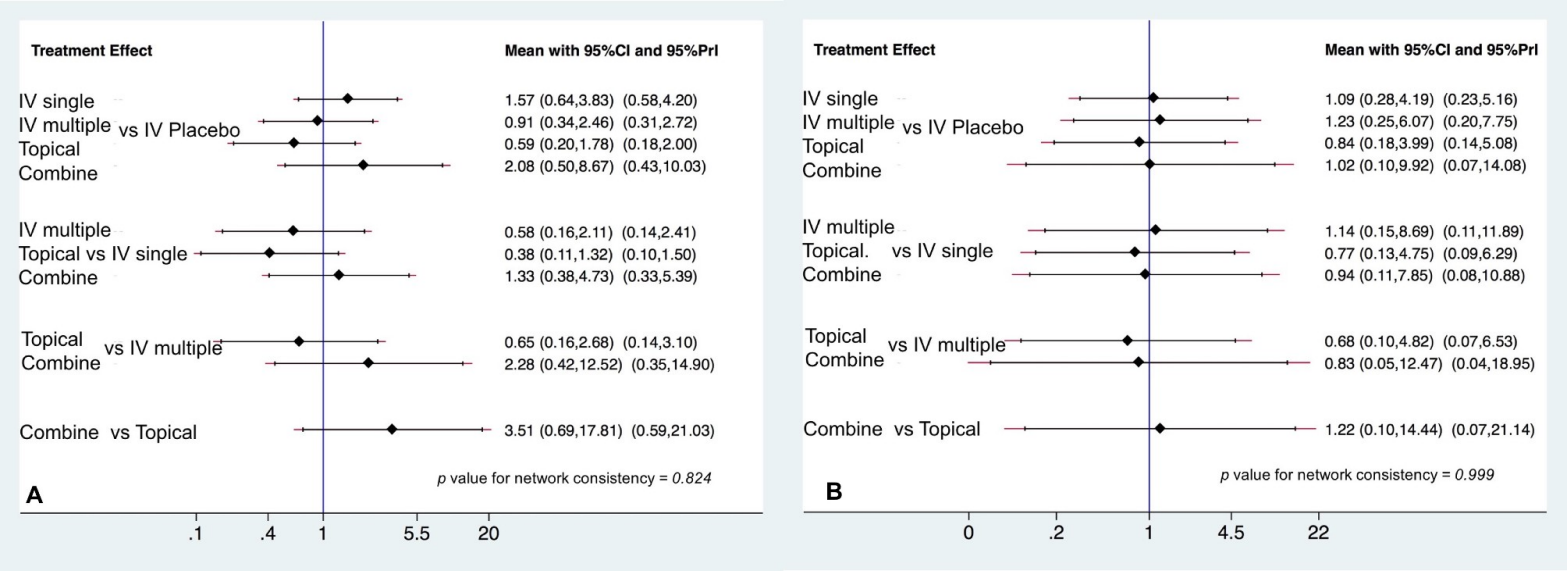
The interval plot of the odds ratio for the safety by (A) deep vein thrombosis and by (B) pulmonary embolism.

A total of 23 trials[17–23, 25, 27–41] with 1982 patients reported the data for DVT, and 18 trials[17–21, 23, 25, 27, 28, 30, 32–34, 36–40] with 1776 patients reported data for PE. In this analysis, placebo was used as the reference. No significant difference for the rate of DVT occurrence was observed for IV single (OR = 1.57, 95% CI: 0.64–3.83, p = 0.32); IV multiple (OR = 0.91, 95% CI: 0.34–2.46, p = 0.86); topical use (OR = 0.59, 95% CI: 0.20–1.78, p = 0.35); or combined use (OR = 2.08, 95% CI: 0.50–8.67, p = 0.31) compared with placebo; the results were the same for PE (Fig 5). The ranking of the five interventions with regards to total blood loss and drain out is summarized in Fig 4.

### Sensitivity analysis

The result of the sensitivity analysis, which included only studies in which transfusions were performed with a strict transfusion strategy or excluded two studies that used the “single 3 g” and “triple 3 g” routes, or excluded each one study that included combined treatment was similar to the results of our primary overall results. Odds ratios, standardized mean differences, and rankings did not change considerably.

### Literature quality assessment and publication bias

In terms of the methodological quality, subjects were randomized using an established allocation sequence, and the investigators and research assistants were blinded to the allocation. However, it is unclear whether the trials that were included meet all of the quality assessment criteria (S2 Fig). Based on the adjusted funnel plot there was no evidence of publication bias for the set of studies related to each results (S3 to S7 Figs).

## Discussion

The use of TXA in joint arthroplasty is becoming more prevalent for minimizing perioperative blood loss and the need for transfusion because it has been shown to provide both clinical and cost-saving benefits.[42] Surgeons may choose to use TXA intravenously, topically, or a combination of the two. Recently, many meta-analyses have been performed to analyze which route of use for TXA is most beneficial. This has not been fully clarified yet because the previous meta-analyses conducted a pair-wise comparison based only on direct evidence. To our knowledge, this is the first attempt to integrate all of the evidence regarding different routes in TXA use for THA in an NMA.

A combined methodology for TXA treatment (IV and topical) is an emerging method for enhancing the hemostasis effects of TXA when performing total joint arthroplasty.[20] Our meta-analysis indicated that the combined use of TXA resulted in a significantly reduced transfusion rate compared with IV or topical use alone. This outcome concurs with a recent meta-analysis that showed combined IV and topical TXA as being more effective for hemostasis compared with IV alone; however, this analysis included only eight RCTs with a total of 850 patients.[6] Our study combined direct and indirect evidence from 25 RCTs involving 2227 patients who underwent THA to estimate the relative efficacy of all routes of TXA.

In our NMA, ‘IV multiple’ use reduced total blood loss compared with IV single, but the difference was not significant. This result suggests that despite greater efficacy with increased dosage, doses in excess of the effective dose confer no significant additional benefit.[43] In high risk patient, such as patients with upper gastrointestinal bleeding, they may use up to 4 grams per day.[44] But even in case of drug with high therapeutic indexes, clinicians should strive to administer the minimum dose to reach a desired goal. Depending on the expected degree of bleeding and the patient’s condition, it may be possible to consider using different routes.

Our results showed that the IV single, IV multiple, and topical treatments were equivalent for all outcome measurements. We paid special attention to whether the effect of topical TXA has a greater effect on total drain out than total blood loss, although the doses used were comparable to those of IV use. However, there was no significant difference between the two groups with respect to the total drain out. It is suggested that there was no effect on the intraoperative blood loss because TXA delayed lysis of the fibrin clot by the proteolytic action of the plasmin, rather than by influencing primary hemostasis and coagulation.[27]

There were no significant differences across all interventions, including placebo, for incidence of DVT and PE, thus confirming the safety of TXA use. Topical application, or small doses of TXA, are used to avoid systemic complications. TXA is known to not suppress the fibrinolytic activity in normal vessel walls, which is the most important link in the fibrinolytic defense system.[45] This is the most potent reason why IV TXA will not increase the occurrence of DVT after THA. All of the RCTs that we evaluated excluded high-risk patients for thrombosis, such as those with a history of ischemic heart disease, cerebrovascular disease, or thromboembolic disease. Thus, it is necessary to confirm whether combined TXA is safe in patients who are susceptible to these types of events, although our study demonstrated that TXA safely reduces the transfusion rate without thromboembolic risk.

Our study was carefully designed to reduce bias, but several limitations remain. First, with regards to the TXA, the variation in regimens between the studies might cause heterogeneity. To clarify this issue, we classified IV use into IV single and IV multiple. Second, differences in the transfusion criteria, surgical approaches, and postoperative blood saving measures, patient age, as well as the protocols for thromboprophylaxis may have influenced the results. However, the OR values are meaningful as they have been calculated using the same protocol within each study.

In summary, compared with topical and IV TXA use, combined TXA treatment can decrease the transfusion requirement without an increased risk of complications (DVT and PE). The topical administration alone of TXA did not decrease total drain out and had a similar effect when compared with IV administration. Surgeons might consider using combine TXA treatment when there may be tendency for bleeding or a difficult surgery. However, it has not been confirmed that this is safe for patients who are susceptible to DVT or PE. Randomized trials are needed to clarify the optimal dosing and time to administration of TXA.

## Supporting information

S1 Table Updated checklist for network meta-analysis of randomized clinical trials. (DOCX)

S2 Table The search strategy that details the searching process of relevant clinical study selection. (DOCX)

S1 Fig Contributions matrix: percentage contribution of each direct estimate to the network meta- analysis estimates for preventing blood transfusions. In these matrices, the size of each square is proportional to the weight attached to each direct summary effect. A: IV single B: IV multiple C: topical D: combined E: placebo.
(TIFF)

S2 Fig Risk of graph: review authors’ judgements about each risk of bias item presented as percentages across all included studies. (TIFF)

S3 Fig Ajusted funnel plot for network meta-analysis of need for blood transfusion. (TIF)

S4 Fig Ajusted funnel plot for network meta-analysis of total blood loss. (TIF)

S5 Fig Ajusted funnel plot for network meta-analysis of total drain out. (TIF)

S6 Fig Ajusted funnel plot for network meta-analysis of deep veion thrombosis. (TIF)

S7 Fig Ajusted funnel plot for network meta-analysis of pulmonary embolism. (TIF)
