# Supplementary material for: Optimal use of tranexamic acid for total hip arthroplasty: A network meta-analysis
Source: PLoS One. 2018 Oct 31;13(10):e0206480. doi: 10.1371/journal.pone.0206480 (PMC6209331; doi:10.1371/journal.pone.0206480)
Supplement: S2 Table — (DOCX) [file pone.0206480.s002.docx]

**Queries**

1. Population
   Patient who underwent hip arthroplasty or replacement
2. Intervention
   Tranexamic acid
3. Study type

Randomized controlled trial

**Pubmed 20170403 – 459 articles**

Search strategy on medline

#1: Arthroplasty [Mesh terms]

#2: Arthroplasty, Replacement, Hip [Mesh terms]

#3: Arthroplasty [All field]

#4: Total hip arthroplasty [All field]

#5: Hemiarthroplasty [Mesh terms]

#6: Hemiarthroplasty [All field]

#7: Prosthetic Replacement [All field])

#8: #1 OR #2 OR #3 OR #4 OR #5 OR #6 OR #7

#9: Tranexamic acid [Mesh]

#10: Tranexamic acid [All field]

#11: Tranexamic[All field])

#12: #9 OR #10 OR #11

#13: Randomized controlled trial [pytp]

#14: #8 AND #12 AND #13

**
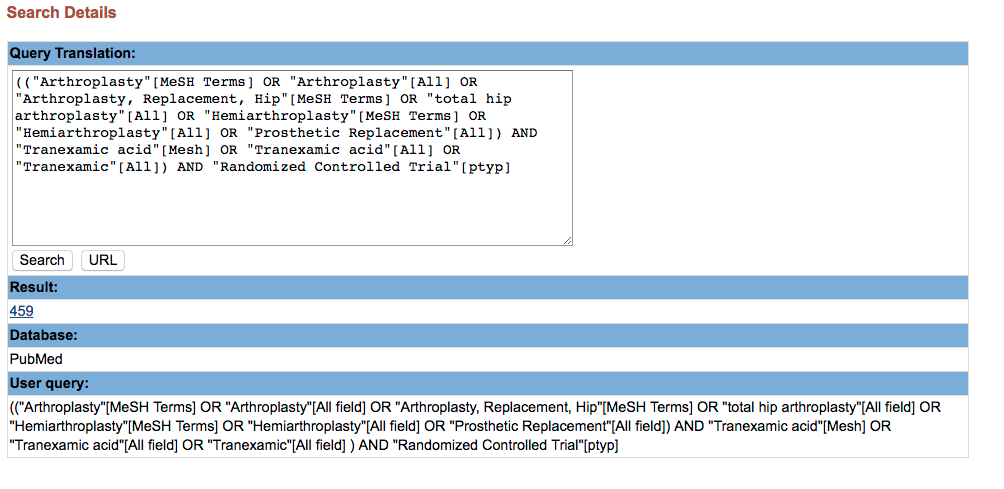
**

**Cochrane 20170403 – 135 articles**

(“Arthroplasty, Replacement, Hip”[MeSH Terms] OR “Hip arthroplasty” OR “Hip Replacement” OR “Hemiarthroplasty”) AND (“Tranexamic acid”[Mesh] OR “Tranexamic acid” OR “Tranexamic”)


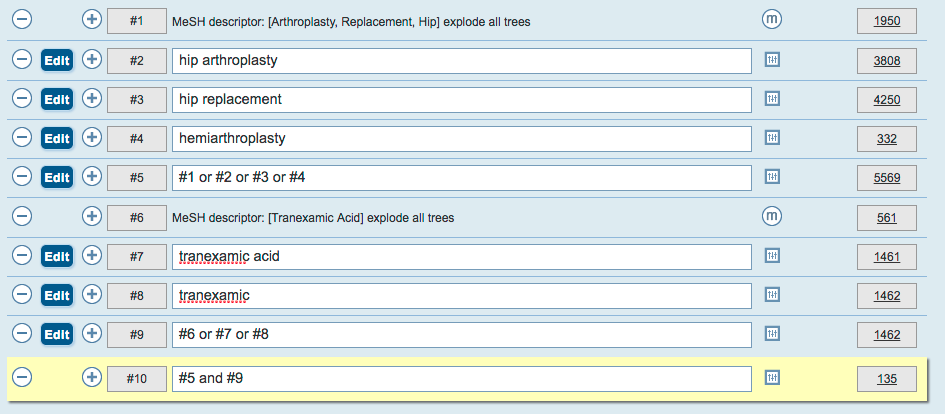


21 reviews and 113 trials

**EMbase 20170403 - 322 articles**

Emtree term: ‘hip arthroplasty’/exp , 'tranexamic acid'/exp

'hip arthroplasty'/exp OR 'hip arthroplasty' OR 'hip replacement' AND ('tranexamic acid'/exp OR 'tranexamic acid' OR 'tranexamic')
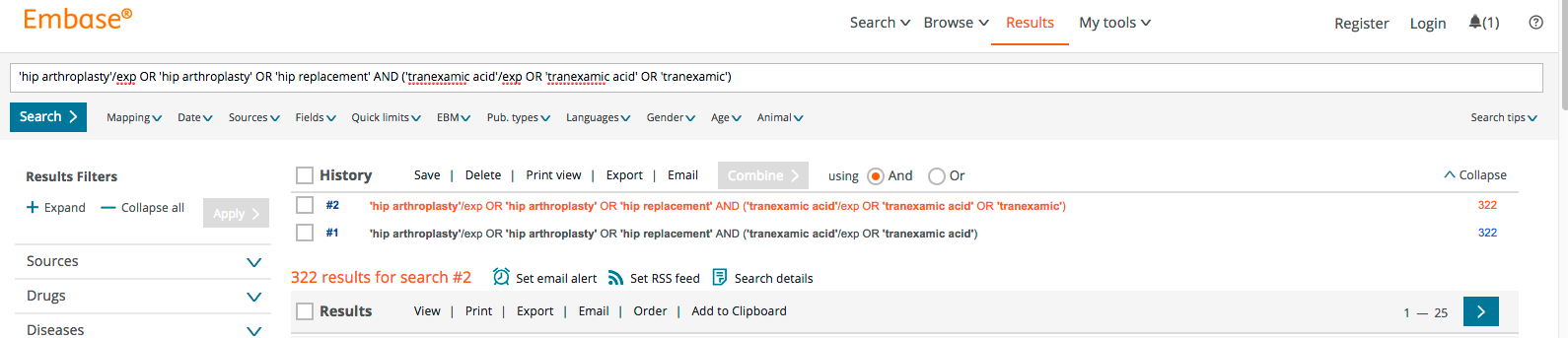


**Hand searching – 5 articles**
