# Supplementary figures and images for: Optimal use of tranexamic acid for total hip arthroplasty: A network meta-analysis
Source: PLoS One. 2018 Oct 31;13(10):e0206480. doi: 10.1371/journal.pone.0206480 (PMC6209331; doi:10.1371/journal.pone.0206480)

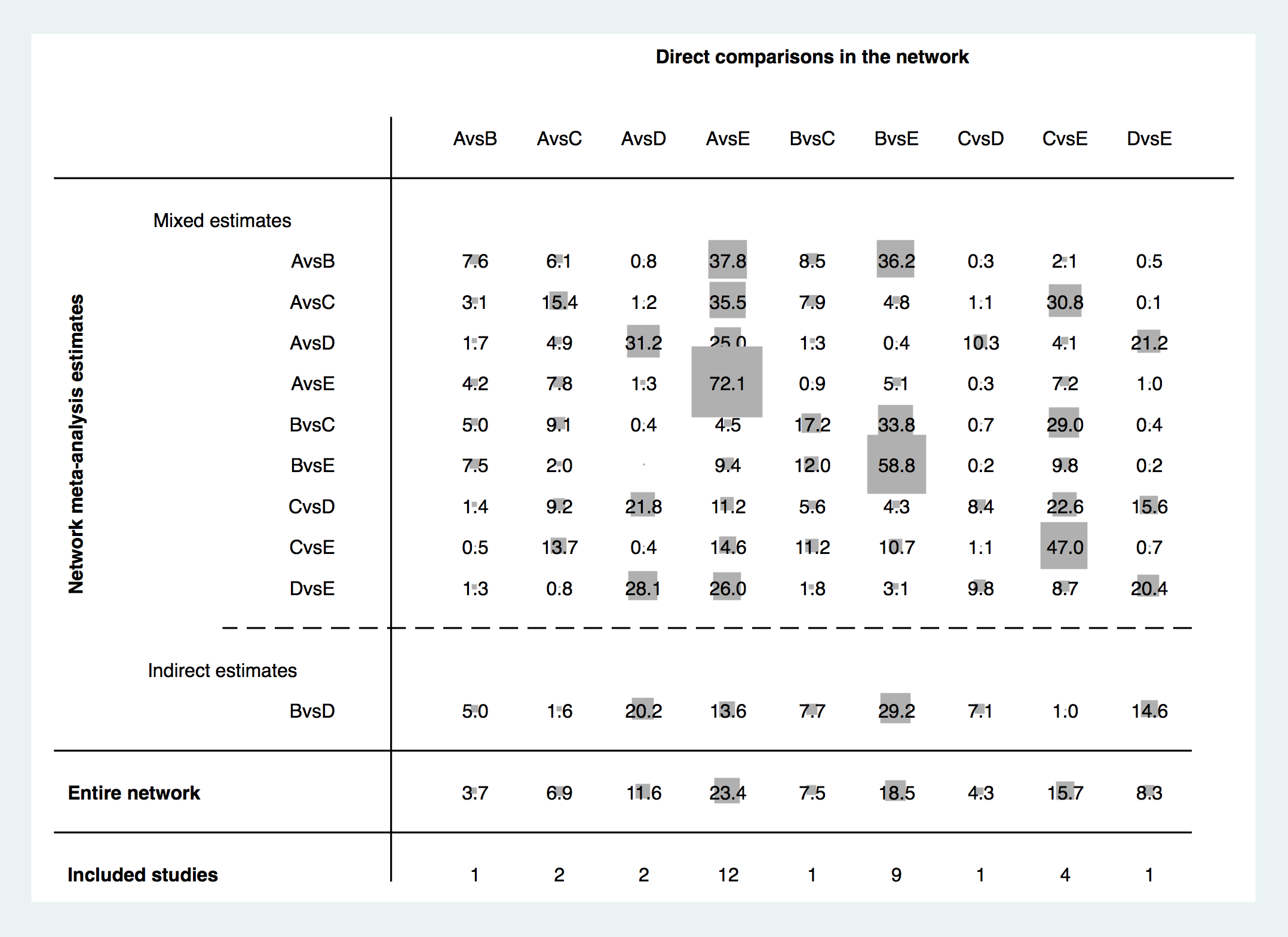

Supplement: S1 Fig — In these matrices, the size of each square is proportional to the weight attached to each direct summary effect. A: IV single B: IV multiple C: topical D: combined E: placebo. (TIFF) [file pone.0206480.s003.tiff]

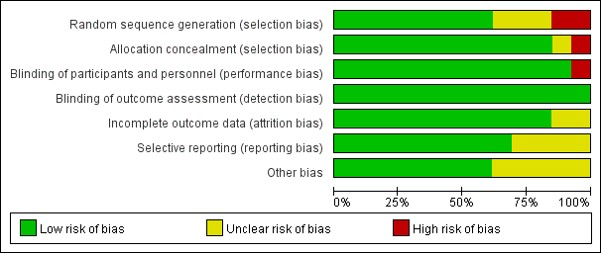

Supplement: S2 Fig — (TIFF) [file pone.0206480.s004.tiff]

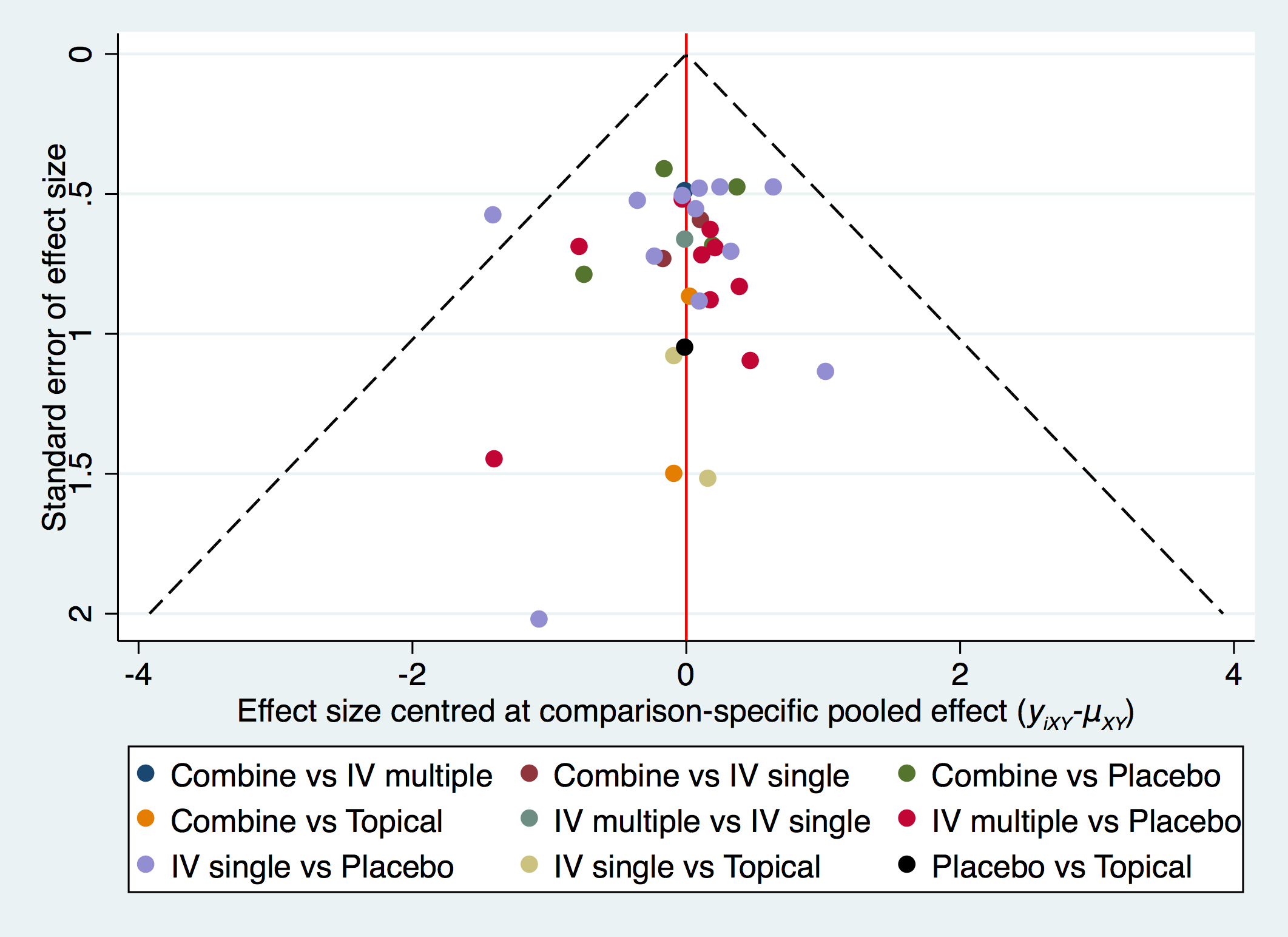

Supplement: S3 Fig — (TIF) [file pone.0206480.s005.tif]

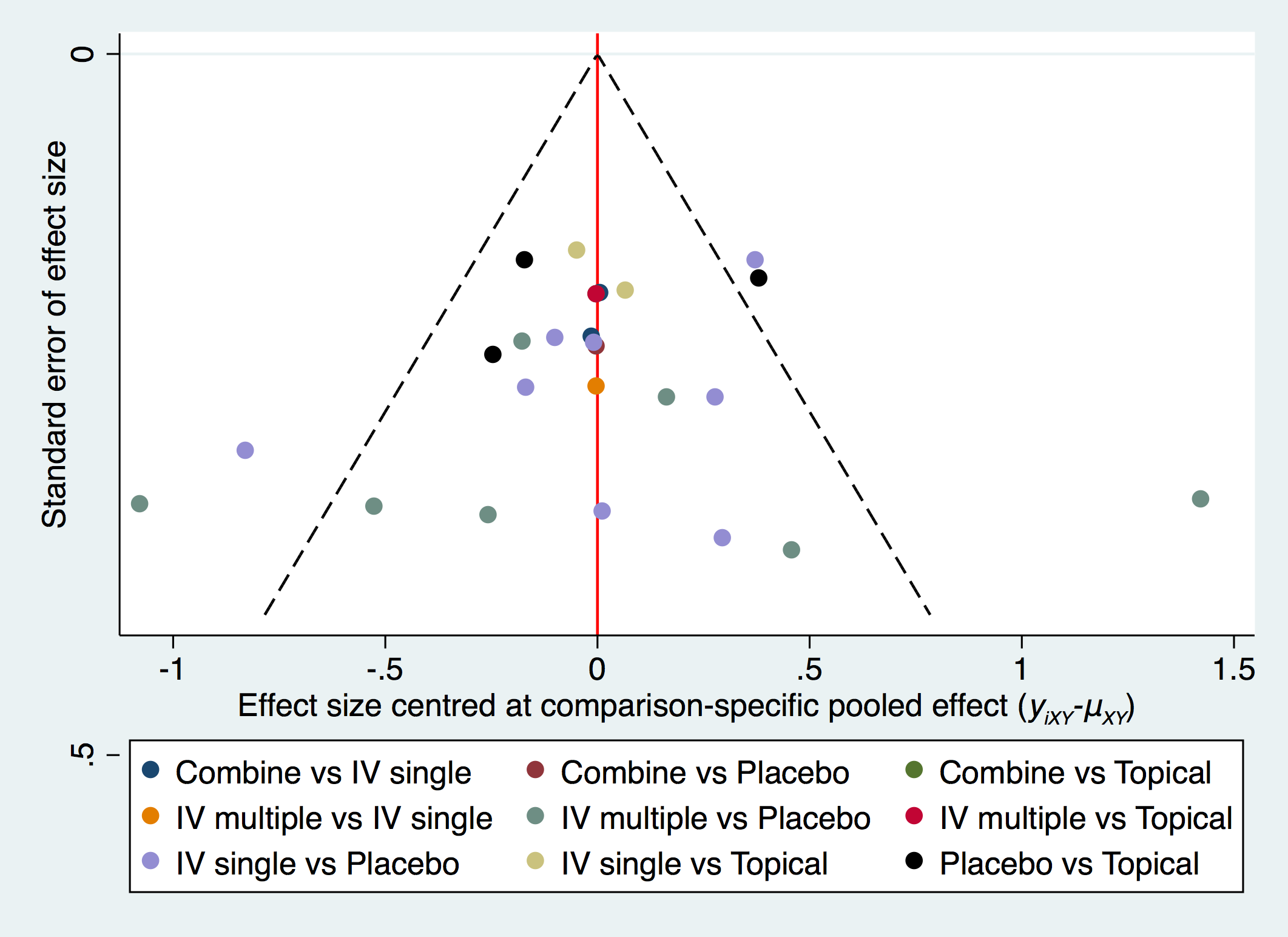

Supplement: S4 Fig — (TIF) [file pone.0206480.s006.tif]

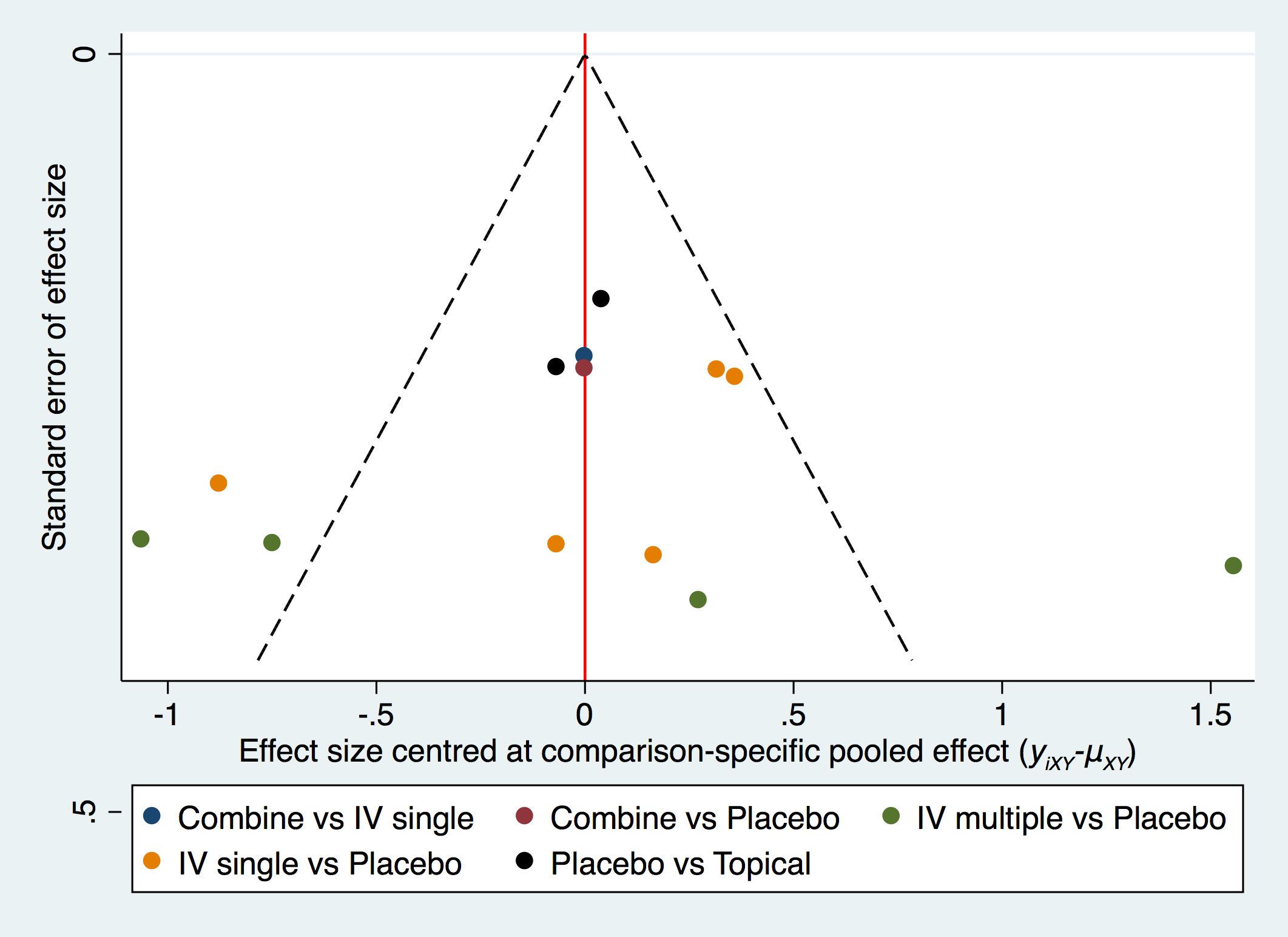

Supplement: S5 Fig — (TIF) [file pone.0206480.s007.tif]

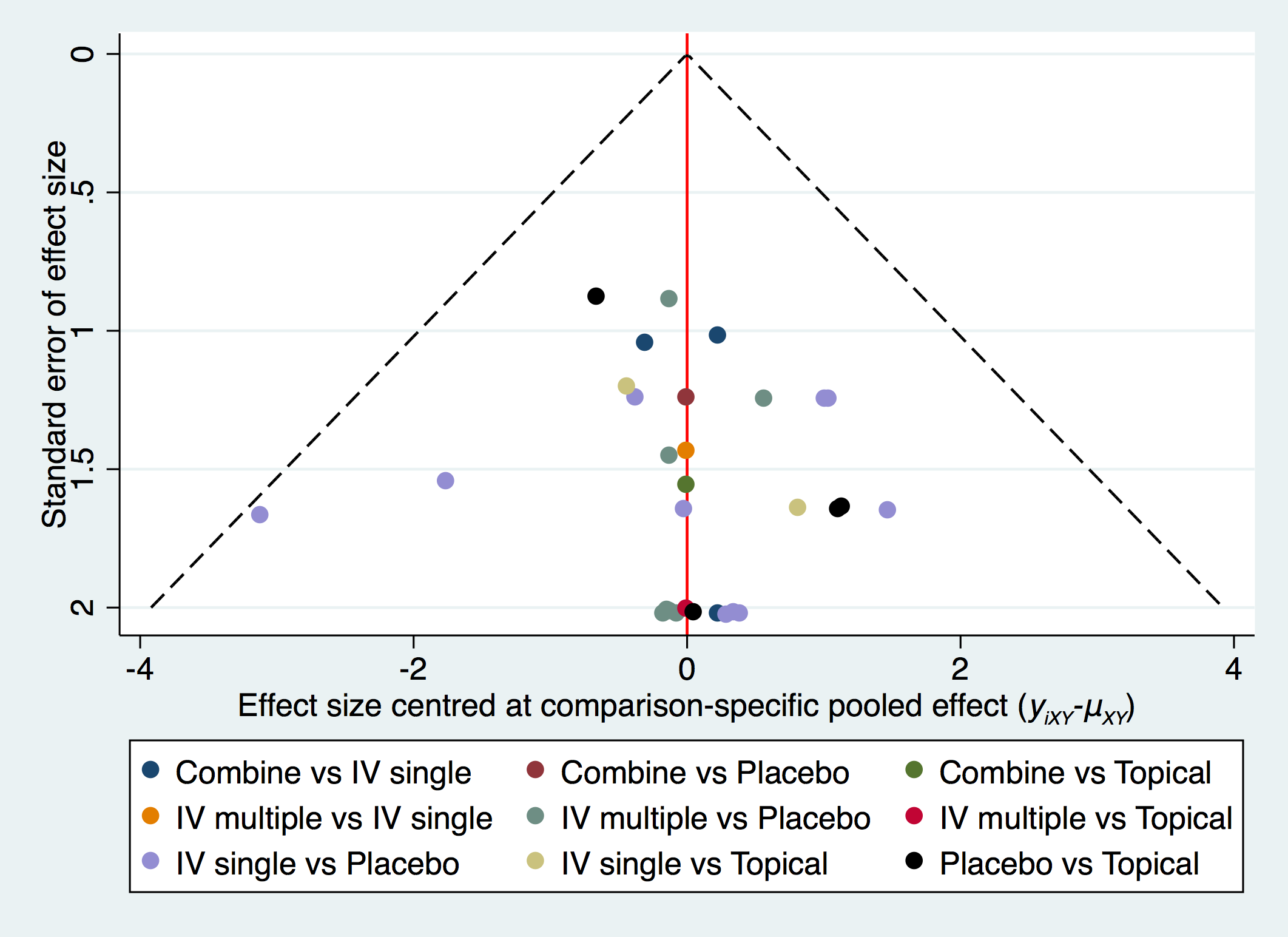

Supplement: S6 Fig — (TIF) [file pone.0206480.s008.tif]

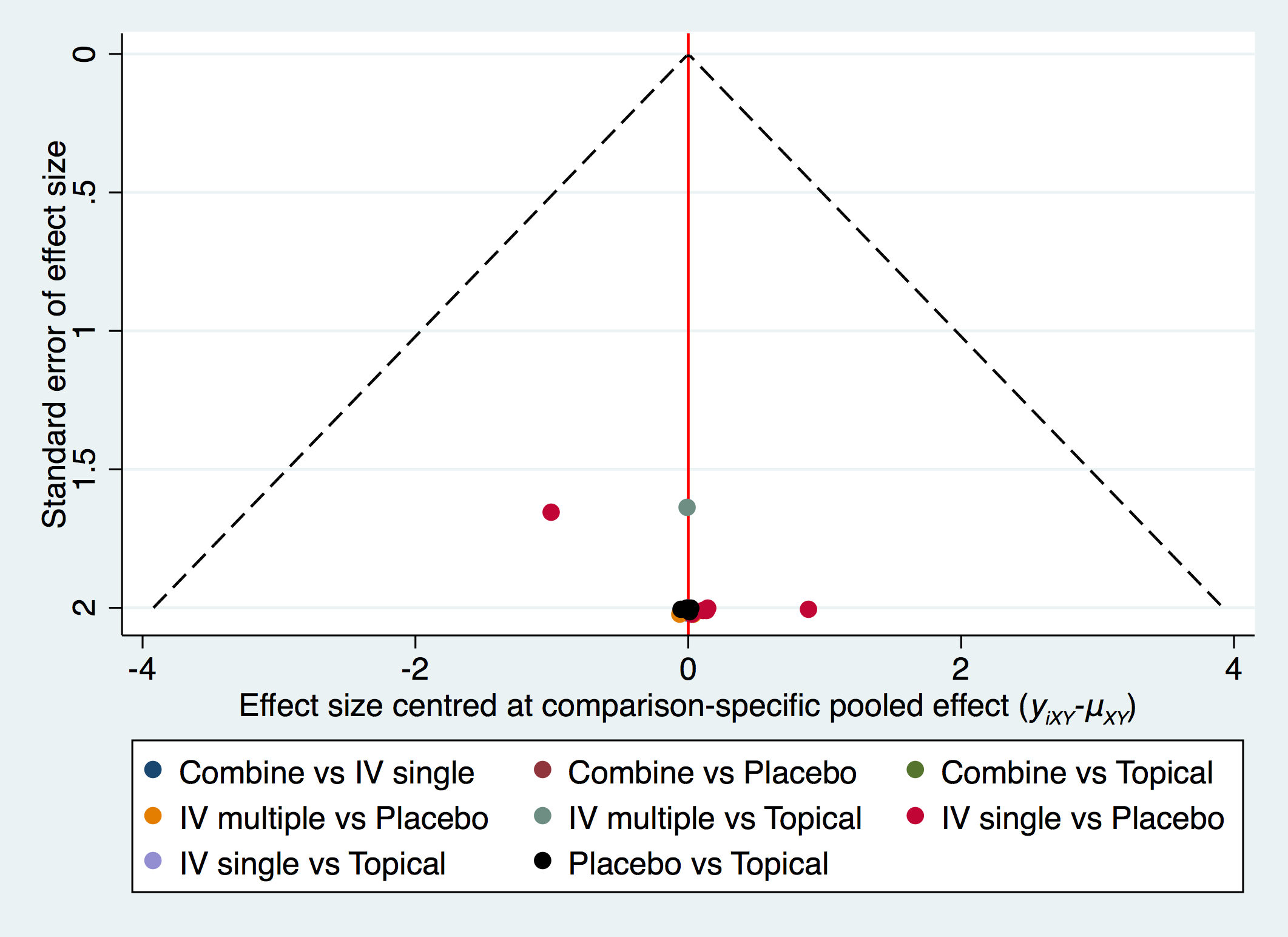

Supplement: S7 Fig — (TIF) [file pone.0206480.s009.tif]
